# Distinct Role of Rab27a in Granule Movement at the Plasma Membrane and in the Cytosol of NK Cells

**DOI:** 10.1371/journal.pone.0012870

**Published:** 2010-09-21

**Authors:** Dongfang Liu, Tobias Meckel, Eric O. Long

**Affiliations:** Laboratory of Immunogenetics, National Institute of Allergy and Infectious Diseases, National Institutes of Health, Rockville, Maryland, United States of America; University of Sheffield, United Kingdom

## Abstract

Protocols were developed to automate image analysis and to track the movement of thousands of vesicular compartments in live cells. Algorithms were used to discriminate among different types of movement (e.g. random, caged, and directed). We applied these tools to investigate the steady-state distribution and movement of lytic granules (LG) in live natural killer (NK) cells by high-speed 3-dimensional (3D) spinning disc confocal and 2-dimensional total internal reflection fluorescence microscopy. Both mouse NK cells and a human NK cell line deficient in the small GTPase Rab27a were examined. The unbiased analysis of large datasets led to the following observations and conclusions. The majority of LG in the cytosol and at the plasma membrane of unstimulated NK cells are mobile. The use of inhibitors indicated that movement in the cytosol required microtubules but not actin, whereas movement at the plasma membrane required both. Rab27a deficiency resulted in fewer LG, and in a reduced fraction of mobile LG, at the plasma membrane. In contrast, loss of Rab27a increased the fraction of mobile LG and the extent of their movement in the cytosol. Therefore, in addition to its documented role in LG delivery to the plasma membrane, Rab27a may restrict LG movement in the cytosol.

## Introduction

Cytotoxic T cells and natural killer (NK) cells kill virus-infected cells and tumor cells [1]. Both T and NK cells kill target cells through polarized exocytosis of lytic granules (LG, also called secretory lysosomes), which contain death-inducing proteins (cytolytic effector molecules) such as perforin, granzymes, and Fas ligand [2], [3], [4]. Granule exocytosis involves polarization towards the immunological synapse, docking at the plasma membrane (PM), and fusion with the PM [5]. A dynamic actin cytoskeleton is indispensable for target cell killing by cytotoxic T lymphocytes and NK cells [6], [7], [8], [9]. Cytoskeletal dynamics and polarization during NK cell cytotoxicity provides a series of checkpoints [10]. Little is known about the movement of LG at steady-state in unstimulated cytotoxic lymphocytes. Even in the absence of NK cell stimulation by target cells or by cell surface receptors, some of the LG are close to the PM, as visualized by total internal reflection fluorescence (TIRF) microscopy [11], [12]. LG that are close to the PM may represent a functional pool available for release of cytolytic effectors, as degranulation by NK cells has been observed in the absence of granule polarization [13], [14].

Rab27a, a Ras-like GTPase protein, is defective in patients with Griscelli Syndrome type 2, which is caused by mutants in the *RAB27A* gene [15]. Griscelli syndrome type 2 (OMIM no. 607624) is an autosomal recessive, rare immune disorder associated with hypopigmentation [15]. Rab27a defects are also associated with impaired cytotoxicity [16], [17] and with poor docking of LG at the PM, as shown by electron microscopy in fixed cells [18]. However, the two *RAB27A* missense mutations (K22R and I44T) do not confer a dominant negative function on Rab27a in melanosome transport [19]. The corresponding mouse model is Rab27a^ash^ mice [20]. Rab27a mutant mice (*ashen* mice), lack the Rab27a protein [21], as the mutant transcripts are nonfunctional [20]. The lack of Rab27a in *Ashen* mice causes a defect in vesicle tethering to the PM, as well as in exocytosis of LG [18], [20], [22]. Here we examined how Rab27a regulates LG movement both at the PM and in the cytosol of human and of mouse NK cells in the absence of activation signals.

We used high-speed spinning disc confocal microscopy for 3D single-granule tracking in the cytosol and TIRF microscopy for 2D single-granule tracking at the PM to investigate LG movement. Automated image analysis allowed us to precisely quantify and characterize the movement of thousands of individual LG and hence perform robust statistical analysis of large data sets. A human NK cell line with stable knock-down of Rab27a and primary NK cells from Rab27a-mutant mice were used to study the role of Rab27a in LG mobility. We found that the majority of LG in the cytosol and at the PM of unstimulated NK cells are mobile. As expected, fewer LG reached the PM in the absence of Rab27a. Analysis of LG movement revealed that Rab27a has a different effect at the PM and in the cytosol. Whereas Rab27a enhances movement of LG at the PM, it constrains their movement along microtubule (MT) inside the cells.

## Results

### Rab27a Defect Reduces the Number of LG at the Plasma Membrane

**Figure 1:**
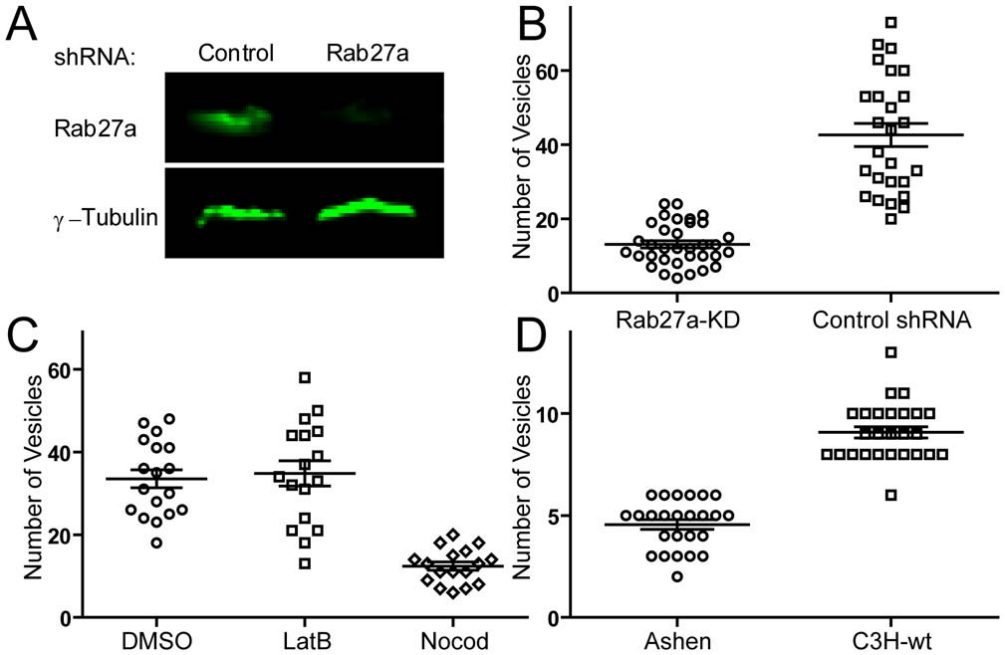
Knockdown of Rab27a and disruption of microtubules decreased the number of granules docked at the PM. (A) Knockdown of Rab27a expression in NKL cells. The shRNA against Rab27a was stably transfected into NKL cells. Rab27a was detected by Western blot and visualized by Odyssey Infrared Imaging (Li-Cor Biosciences). (B) Number of granules within evanescent filed per NKL cell between control shRNA and Rab27a knockdown (Rab27a-KD) visualized by TIRF microscopy. (C) Number of granules within evanescent filed per NKL cell pretreated with 10 µM Latrunculin B (LatB) and Nocodazole (Nocod). (D) Number of acidic compartments within evanescent filed per NK cell from *Ashen* (Rab27a mutation) and C3H (wild-type) mice. The data are representative of at least 50 cells in three independent experiments.

The NK cell line NKL was transfected with GFP-FasL (GFP fused to the N-terminal, cytosolic tail of FasL) in order to visualize LG [4]. NKL-GFP-FasL cells were re-transfected with a plasmid encoding shRNA against Rab27a, and clones with stable shRNA expression were isolated. Expression of Rab27a was monitored by Western blot analysis (Fig. 1A). In stably transfected NKL-GFP-FasL cells, most of the GFP-FasL-labeled granules contained perforin, as detected by intracellular staining of perforin (data not shown). The Pearson correlation coefficient was 0.78±0.01 (n = 11) in control cells. Knock-down of Rab27a expression did not change the sorting of perforin and FasL, as their colocalization was still observed, with a Pearson correlation coefficient of 0.77±0.02 (n = 10).

The distribution of LG was investigated by TIRF microscopy (TIRFM). TIRFM has been widely used to track the movement of single vesicles within cells. TIRFM provides several advantages for imaging processes in living cells, such as low background, high temporal resolution, minimal photobleaching, and minimal phototoxicity, which have made it a powerful tool to study granule movement and fusion with the PM [23], [24], [25], [26]. The evanescent field generated by TIRF illumination penetrates only ∼200 nm from the coverslip in our microscope setup. Under these conditions, LG labeled by expression of GFP-FasL were easily observed in close proximity to the PM. As shown in Video S1, the majority of LG at the PM are mobile. Knockdown of Rab27a (Rab27a-KD) decreased the number of LG per cell contact detectable within the evanescent field from 42.6±3.1 (shRNA control, n = 26) to 13.1±0.95 (Rab27a-KD, n = 34) (Fig. 1B and Video S1). While Latrunculin B (LatB) treatment did not change the number of LG, depolymerization of MT by Nocodazole (Nocod) treatment significantly decreased the number of LG (Fig. 1C). No noticeable difference of the contact area to the poly-L-lysine-coated coverslip has been observed between untreated and inhibitor-treated cells, as well as between shRNA control and Rab27a-KD NKL cells (data not shown). Similar results were obtained from Rab27a mutant mice (*Ashen*) as compared to wild-type mice (C3H) (Fig. 1D). The LG of mouse NK cells are secretory lysosomes [27], [28] which, as all acidic compartments, can be labeled with LysoTracker Red DND-99 [29], [30] and imaged by TIRFM. The number of LG per cell contact decreased from 9.1±0.27 (C3H, n = 26) to 4.5±0.23 in the absence of Rab27a (*Ashen*, n = 25). Therefore, fewer LG reside near the PM in the absence of Rab27a, consistent with a role of Rab27a in transport to, or retention at the PM [20].

### Rab27a Enhances Cytoskeleton Dependent Movement of LG at the PM

**Figure 2:**
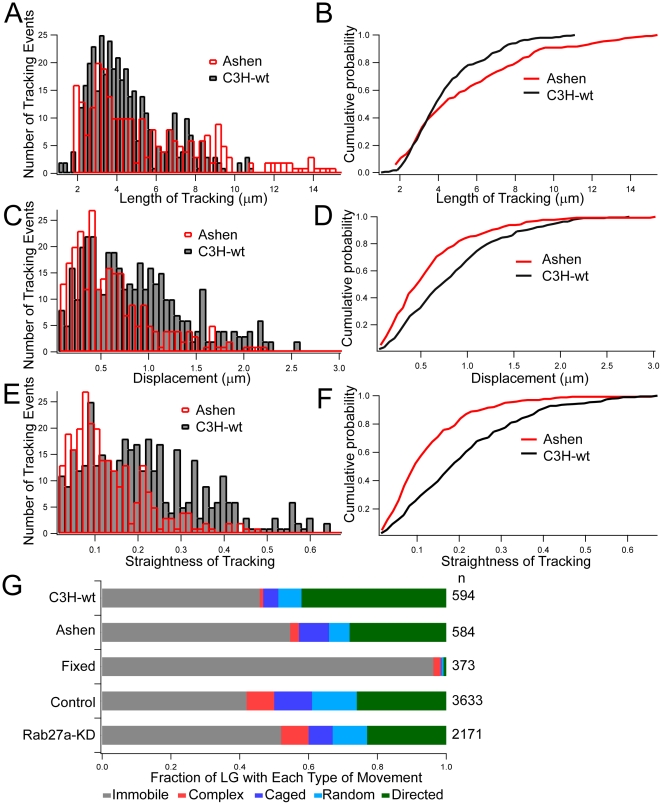
Rab27a mutant decreased straightness and displacement but increased length of LG tracking at the PM. The length (A and B), displacement (C and D), and straightness (E and F) of LG trajectories from *Ashen* (red) and CH3 mice (black) are compared using histograms and cumulative probability plots. The data is derived from granules that travel within 10 seconds. The relative occurrence of each type of movement for each indicated condition is summarized in a bar plot (G). The numbers of tracking events (n) are listed on the right. The data are representative of at least two independent experiments.

Several parameters of LG movement were determined by automated image analysis of TIRF data to evaluate how Rab27a may affect granule movement in mouse NK cells. The length of tracking paths at the PM increased in Rab27a-mutant NK cells from *Ashen* mice (Fig. 2A and 2B), but displacement and straightness decreased (Fig. 2C–2F). The Rab27a mutation decreased the fraction of LG with directed movement from 42% (C3H wild-type, n = 594, in 27 cells) to 28% (*Ashen*, n = 584, in 24 cells). Similar results were obtained with Rab27a knockdown in NKL cells (Fig. 2G). Comparable results were observed with two independent Rab27a knockdown NKL clones (data not shown). Consistent with previous studies [18], [22], [31], NK cells lacking Rab27a contain acidic compartments of apparently normal size, number, and morphology (data not shown). These data imply that Rab27a enhances the movement of LG that are close to the PM.

To test how the actin cytoskeleton and MT affect LG movement at the PM, NKL cells were treated with different concentrations of LatB and Nocod. LatB alone, Nocod alone, and the combination of LatB and Nocod (LatB + Nocod) decreased displacement and straightness of movement (Figure S1). Therefore, LG movement close to the PM is dependent on both actin and MT.

We further analyzed how disruption of the cytoskeleton changed the fraction of LG within each type of movement. LatB alone, Nocod alone, and LatB + Nocod treatment increased the fraction of immobile LG and decreased the fraction of LG with directed movement (Figure S2). LatB alone increased the fraction of immobile LG from 42% (Control, n = 3633) to 86.9% (LatB treatment, n = 2013), as did Nocod alone (87.2%, Nocod treatment, n = 1611) and LatB + Nocod (93.4%, LatB + Nocod treatment, n = 1529). LatB and Nocod each decreased the fraction of LG with directed movement from 26% (Control) to 8% (LatB alone or Nocod alone treatment), as did the combination of LatB and Nocod (4%, n = 1529). Therefore, the actin cytoskeleton and MT are required for the movement of LG at the PM. We conclude that F-actin, at physiological concentrations, promotes LG movement at the PM. Rab27a does not promote arrest (or stable docking) at the PM, but enhances actin- and MT-dependent directed granule movement at the PM.

### The Cytoskeleton Regulates LG Dynamics in the Cytosol

**Figure 3:**
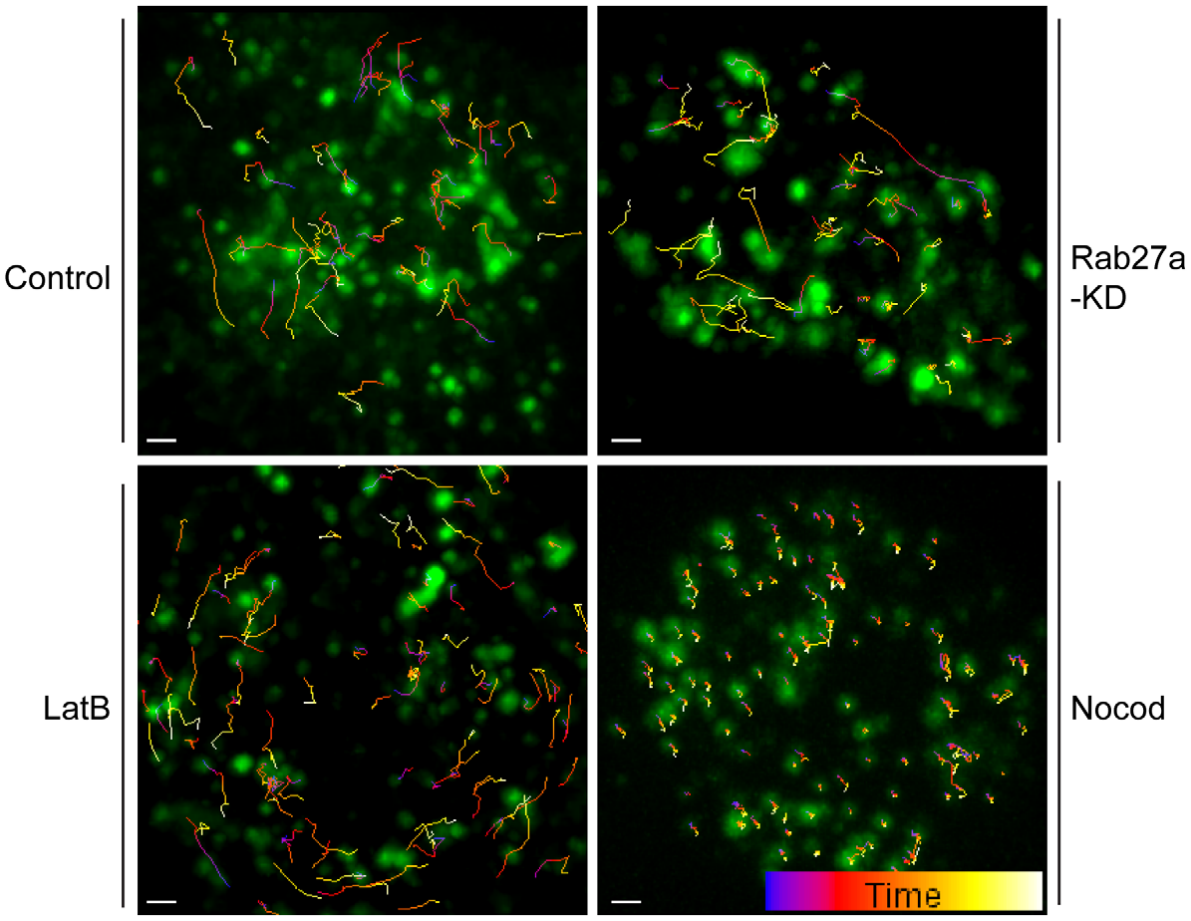
3D movement of single LG tracked in live NK cells. Single vesicle tracks are superimposed on maximum intensity projections of image stacks as used for the tracking analysis (see Material and Methods). The x-y plane (which is shown) is projected in the z direction. NKL cells were either untreated (control), or treated with 10 µM LatB, 10 µM Nocod, or after Rab27a-KD, as indicated. LG were labeled by expression of FasL-GFP. The colors of tracks indicate the time, according to the color bar (start  =  blue, end  =  yellow). Scale bar is 1.0 µm.

To visualize 3D movement of LG deeper inside the cells, we set up a spinning disc confocal microscopy imaging system capable of recording a 3D data set every second (which is the time for acquiring each 3D stack). At each time point 40 axial steps separated by 0.2 µm in the z-direction were collected into a 3D-stack. Movement of LG was tracked in live, unstimulated NK cells by combining this data set with 3D particle tracking (Fig. 3 and Video S2). A striking observation was that LG in unstimulated NK cells (i.e. in the absence of cytokines or sensitive target cells) are mobile. The proportion of mobile LG, including those with random, directed, caged, and complex movement, was about 70%. After treatment with LatB, LG movement became more peripheral (Fig. 3 and Video S3). However, LatB was not sufficient to promote association of LG with the PM, given that the average number of LG visible by TIRF did not increase (Fig. 1). MT disruption in NKL cells treated with Nocod caused a significant decrease in the length of tracks (Fig. 3 and Figure S3), consistent with the role of MT in LG movement [32], [33]. At a higher concentration (10 µM), Nocod almost completely abolished long-range movement (Figure S3 and Video S4). We further analyzed whether disruption of F-actin and MT affected straightness and track lengths in each condition. LatB did not change the straightness of the tracks (Figure S4A). The track lengths were slightly increased at higher concentrations of LatB treatment (control group: 1.1±0.012 µm, n = 925; 10 µM LatB treatment: 1.3±0.01 µm, n = 547) (Figure S5).

**Figure 4:**
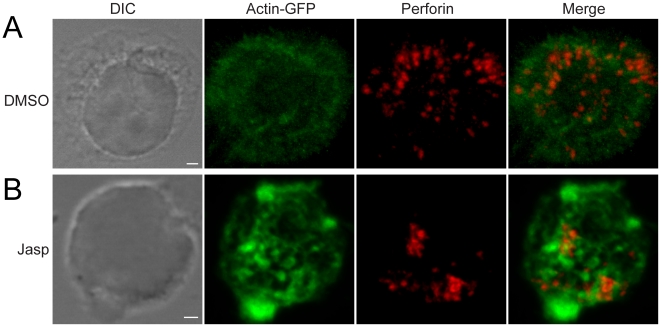
High level of F-actin hinders LG movement in the cytosol. NKL cells were transfected with actin-GFP. Perforin containing LG were stained by anti-perforin monoclonal antibody followed by Alexa Fluor conjugated secondary antibody. The representative images from DMSO vehicle control (A) and 1.0 µM Jasp treatment (B) were shown individually. Scale bar is 2.0 µm. The data are representative of at least three independent experiments.

To further evaluate the role of F-actin in the movement of LG in the cytosol, NK cells were also treated with different concentrations of Jasplakinolide (Jasp), a membrane-permeable compound that binds to and stabilizes actin filaments [34], [35]. Exposure of NKL cells for 30 min to 1.0 µM Jasp resulted in an almost complete inhibition of granule movement (n = 2072 granules in 21 cells) (Figure S4). To further analyze the relationship between F-actin and 3D LG movement, we examined the distribution of actin and perforin containing LG by 3D confocal microscopy. Treatment of NKL with Jasp resulted in much stronger accumulation of actin, as detected by expression of actin-GFP (Fig. 4). In 3D renditions of the data (Video S5 and Video S6) perforin-containing LG appeared to be “caged” within actin-dense regions in Jasp-treated NKL cells. We conclude that high level of F-actin may hinder LG movement in the cytosol.

**Figure 5:**
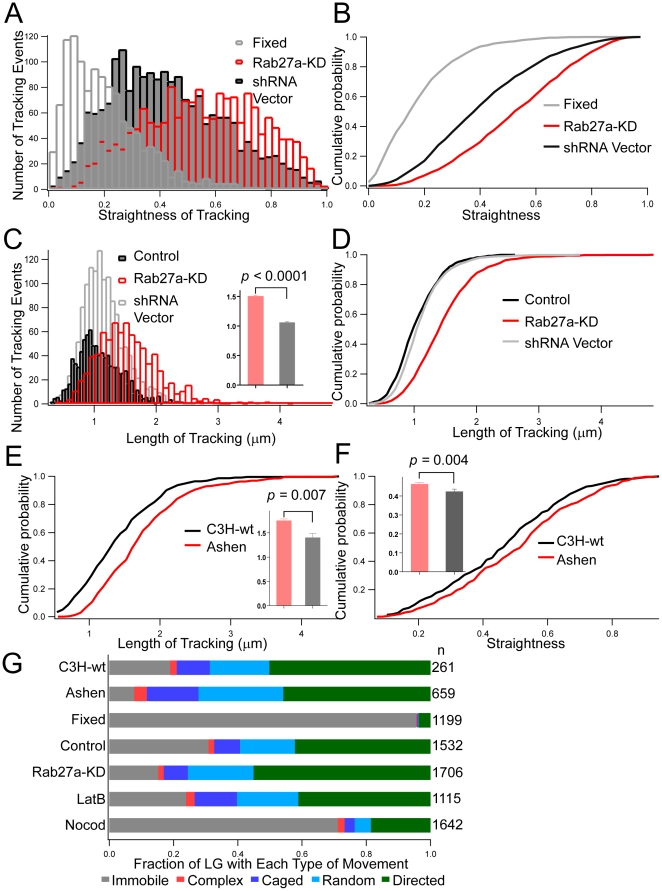
Rab27a defect increased the mobility of cytosolic LG. The straightness of single vesicle trajectories observed in Rab27a knockdown (Rab27a-KD) NKL cells (red), NKL cells expressing control shRNA (control, black), as well as fixed cells (gray) are shown as histograms (A) and cumulative probability plots (B). For the same population of cells, the track lengths are shown as histograms (C) and cumulative probability plots (D). Note that there was no significant difference between control (black) and shRNA vector control (gray) in (C and D). Mean values of track lengths during 10 seconds were compared between Rab27a Knockdown and shRNA vector (C, inset). Finally, length (E) and straightness (F) of single trajectories in NK cells from *Ashen* (red) and C3H mice (black) were compared using cumulative probability plots. Insets show the mean values of track lengths and straightness during 10 seconds between Rab27a mutant mice (*Ashen*, red) and C3H-wt mice (Control, black). The relative occurrence of each type of movement for each indicated condition is summarized in a bar plot (G). The numbers of tracking events (n) are listed on the right. The data are representative of at least two independent experiments.

MT disruption in NKL cells treated with Nocod caused a significant decrease in the straightness, length, displacement, and velocity of tracks (Figure S6). We further investigated how disruption of F-actin and MT affected each type of movement. Disruption of F-actin by LatB did not significantly change the fraction of LG showing directed movement. LatB treatment decreased the fraction of immobile LG from 30.8% (control group, n = 473, from 30 cells) to 22.6% (low concentration of LatB treatment group, n = 338, from 13 cells) or 23.8% (high concentration of LatB treatment group, n = 266, from 21 cells), respectively. However, disruption of MT by Nocod increased the immobilized fraction from 31% (Control) to 71% (Nocod treatment), and decreased the directed movement fraction from 42.2% (Control) to 18.5% (Nocod treatment), respectively (Fig. 5). Therefore, long-range 3D movement in the cytosol requires the integrity of MT but not actin.

### Rab27a Restricts LG Movement in the Cytosol

The effect of Rab27a knockdown on the movement of LG in the cytosol was investigated. Control cells were either untreated NKL cells or NKL cells transfected with control shRNA. No significant difference in LG distribution or movement was observed between these two controls (data not shown). Knockdown of Rab27a did not change the overall distribution of LG in the cytosol (Video S7).We first analyzed whether knockdown of Rab27a affected straightness and track lengths. Knockdown of Rab27a clearly increased the straightness of tracking (Fig. 5A and 5B) and increased the track lengths (Fig. 5C and 5D), as measured in 10 second intervals (shRNA vector control: 1.0±0.01 µm, n = 925; Rab27a-KD: 1.5±0.01 µm, n = 814). To rule out the possibility of variability among separate clones of transfected cells, we generated and analyzed two additional independent clones of Rab27a-KD NKL cells (Rab27a-KD-A1 and Rab27a-KD-A2). Data from these two Rab27a-KD NKL cells were comparable (Figure S7). The effect of Rab27a knockdown on the track lengths in NKL clones was confirmed by analysis of Rab27a-mutant mice (Fig. 5E and 5F). The track lengths in 10 seconds time intervals were significantly increased (C3H wild-type: 1.3±0.09 µm, n = 260; *Ashen*: 1.7±0.04 µm, n = 224) (Fig. 5E). Straightness increased from 0.42±0.01 (C3H wild-type, n = 261) to 0.46±0.007 (*Ashen*, n = 659). Therefore, Rab27a restricts MT-dependent, directed LG movement in the cytosol.

**Figure 6:**
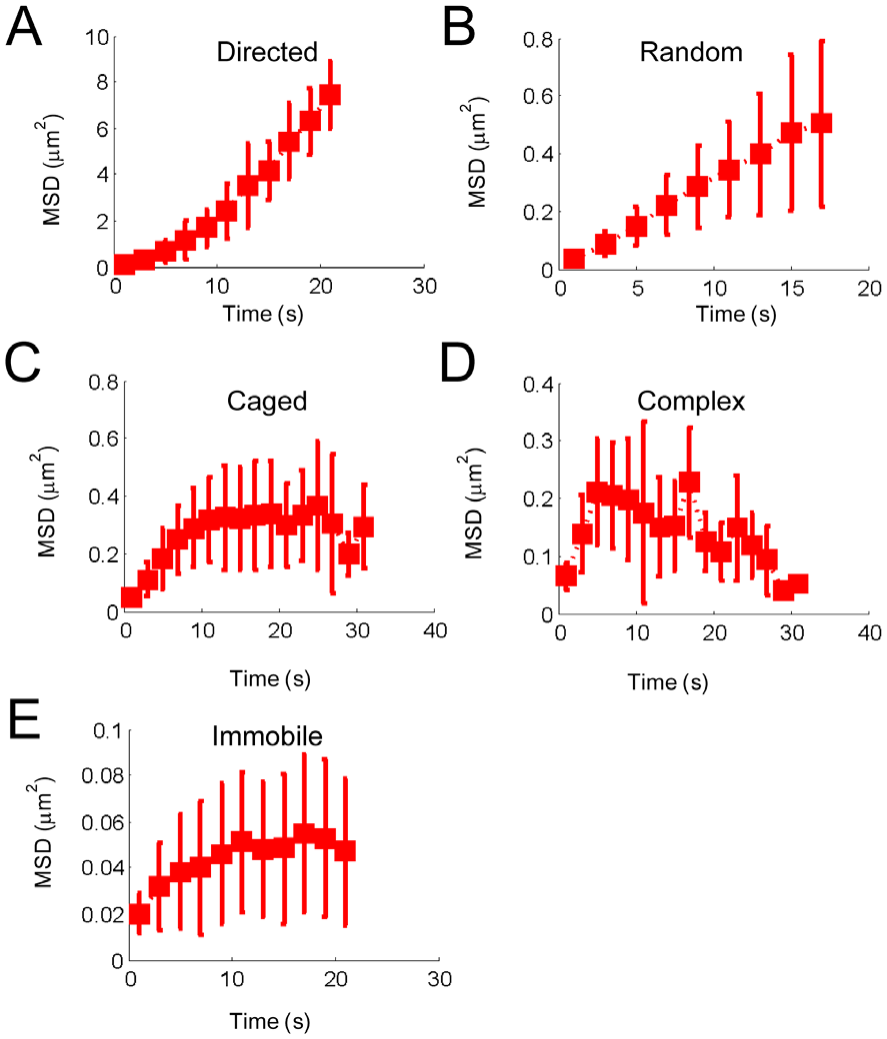
Three-dimensional mean square displacement (MSD) plots of each type of movement. Averages of all 3D-MSD plots from 32 control cells are shown for trajectories showing directed (A), random (B), caged (C), and complex (D) movement, as well as immobile vesicles (E) (see Materials and Methods). Note that the scales among the plots differ.

To further characterize and quantify the mobility of single vesicles, each of the 1532 3D trajectories, which were recorded in 32 control cells (Fig. 5G), was analyzed for its type of movement (i.e. immobilized, random, directed, caged or complex) [36]. The averaged MSD plots for non-treated NK cells are shown in Figure 6, where the characteristic shapes of the MSD plots for, random, directed and caged movement (straight line, upward curvature, downward curvature, respectively) are shown. Knockdown of Rab27a reduced the immobile fraction from 31% (shRNA control, n = 1532) to 15.2% (Rab27a-KD, n = 1706) and increased the directed movement fraction from 42.2% (shRNA vector control, n = 1532) to 55.1% (Rab27a-KD, n = 1706). Knockdown of Rab27a increased the velocity of directed movement (Supplementary Table S1). The velocities of directed movement derived from the MSD fitting are summarized in Supplementary Table S1. Similar results were obtained from Rab27a mutant *Ashen* mice (Fig. 5 and Supplementary Table S1). Therefore, Rab27a plays a role in constraining LG movement along MT in the cytosol.

## Discussion

LG in cytotoxic T lymphocytes from Rab27 mutant *Ashen* mice have a defect in docking at the PM and exocytosis [18], [22]. In this study, we used live imaging of granule movement in unstimulated NK cells and tested the role of Rab27a in controlling movement by knock down in a human NK cell line and by using primary NK cells from Rab27a mutant *Ashen* mice. The movement of individual LG was investigated by high-speed 3D spinning disc confocal and 2D TIRF microscopy in combination with automated image analysis to track several thousands of LG. Applying both imaging techniques allowed us to obtain separate information on LG movement in the cytosol and at the PM. Our data show that while Rab27a enhanced the MT and actin-dependent movement of LG at the PM, it reduced the MT-dependent movement in the cytosol. Therefore, Rab27a has distinct functions in controlling LG movement in the cytosol and at the PM, and exerts its control in unstimulated NK cells in the absence of active granule polarization.

F-actin dynamics is essential for cytotoxicity by T lymphocytes and NK cells [37], [38]. In unstimulated NK cells depolymerization of the actin network by LatB had little effect on granule movement in the cytosol. Therefore, actin is not required for LG movement along MT inside the cells. In contrast, treatment of NK cells with LatB increased the fraction of immobile LG at the PM and reduced the directed movement of mobile LG at the PM. The high level of F-actin induced by Jasp treatment reduced LG movement, consistent with a role of cortical F-actin as a barrier that controls the delivery of LG to the PM in degranulating cells [39], [40], [41]. Whether Rab27a interacts with cytoskeletal elements, thereby influencing LG movement needs to be further investigated.

MT-dependent long-range movement of intracellular organelles is well-documented in neuronal and endocrine cells, in which long-range transport is mediated by MT and short-range transport is mediated by actin [42], [43], [44]. In polarized cytotoxic T cells, LG move along microtubules in a dynein-mediated minus-end movement toward the centrosome, which is pulled towards the cytotoxic immunological synapse [38]. MT integrity is required for NK cell cytotoxic activity [6], [7], [8], [10]. In unstimulated NK cells, Nocod treatment, but not LatB treatment, decreased the velocity, diffusion coefficient, length, and displacement of long-range directed movement of LG in the cytosol. Disruption of MT by Nocod decreased both the number of LG at the PM and the fraction of LG with directed movement at the PM. A pre-docking role for microtubules in the process leading to degranulation in 3T3-L1 adipocytes has been proposed [45]. The loss-of-function of the retinal pigment epithelium Rab27a–Myrip–MyoVIIa complex in *Ashen* mice leads to a reduced constraint on MT-based melanosome motility [46]. Our data demonstrated that movement of LG at the PM of unstimulated NK cells is dependent on both F-actin and MT.

Rab27a is a key component of the vesicle transport machinery in melanocytes [47] and is essential for exocytosis of LG by cytotoxic T lymphocytes [17], [18], [48], of multivesicular endosomes by HeLa cells [49], and of azurophilic granules by neutrophils [50]. Rab27a also plays a role in mediating the tight docking of insulin granules at the PM during glucose stimulation in mouse pancreatic β cells [51]. A mutation in Rab27a causes the defect in the transport of melanosomes in *ashen* mice [20]. Knockdown of Rab27a expression in unstimulated NKL cells and a Rab27a mutation in NK cells from *Ashen* mice resulted in a greatly decreased number of LG at the PM. Although clearly reduced in number as compared to wild-type cells, association of LG with the PM in Rab27a-deficient cells implies that some LG reach the PM stochastically, or that a specific subset of LG reaches the PM by a Rab27a-independent pathway. The defect in LG tethering (or docking) at the PM of Rab27a-deficient cells [18], [22], [52] suggests that Rab27a has a role in the final transport step to the PM and/or retention at the PM. We show here that Rab27a in NKL cells and primary NK cells enhances directed LG movement at the PM, which is consistent with a role of Rab27a in retention, but not stable docking, of LG at the PM. Rab27a increased the fraction of mobile LG at the PM. This could be interpreted either as a direct effect of Rab27a on LG movement at the PM, or as a distinct property of a LG subset (with greater mobility) that is delivered to the PM by a Rab27a-dependent mechanism. To test the latter hypothesis, we carefully analyzed the properties of LG at the PM by calculating the diffusion coefficient of LG in both Rab27a wild-type and Rab27a deficient NK cells. The diffusion coefficient of LG in Rab27a wild-type NK cells is not higher than that of LG in Rab27a deficient NK cells (data not shown), which does not support the existence of distinct subsets of LG. Therefore, this direct effect of Rab27a on LG movement at the PM could occur through its binding to synaptotagmin-like proteins (Slps), which are expressed predominantly at the PM and promote exocytosis of LG in cytotoxic T lymphocytes [53], [54]. A role for Rab27a interaction with Slp1 in anterograde transport of vesicles in axons and exocytosis of neutrophil granules has been reported [55], [56].

Activating signals induce fusion of Rab27a-associated late endosomal vesicles with cytolytic granules in T cells [57], and enhance Rab27a recruitment to LG in NK cells [58]. We report here that Rab27a controls LG movement in the cytosol even in unstimulated cells. The apparent abundance of Rab27a in the cytosol of NKL cells and human resting NK cells, and its association with perforin-containing LG (data not shown), suggests that signal-specific recruitment of Rab27a to LG may not be required for its role in constraining LG movement in the cytosol. NKL cells with reduced Rab27a expression and primary NK cells from Rab27a mutant mice showed an increase in the proportion of mobile LG in the cytosol, implying that Rab27a reduces MT-dependent LG movement.

## Materials and Methods

### Cells, Inhibitors, and Reagents

The human cell line NKL was cultured in RPMI 1640 (Gibco, Grand Island, NY, USA) containing 10% fetal calf serum, 1% L-glutamine, 1% sodium pyruvate, and 100 U/ml of recombinant IL-2 (Hoffmann-La Roche). NKL cells were transfected with a GFP-FasL plasmid (gift of G. Griffiths, Cambridge, UK) by Amaxa nucleofection technology (Amaxa, Cologne, Germany). Briefly, 2×10^6^ NKL cells and 4.0 µg DNA were resuspended in solution, and transfected using nucleofector kit V and program O-17. After transfection, cells were transferred into 6-well plates at 37°C. After 72 hours, 1.0 mg/ml G418 was added. Transfected NKL cells were subcloned to obtain a stable NKL-GFP-FasL cell line. NKL cells were pre-incubated with the following inhibitors for 30 min at 37°C, where indicated: 0.5 and 10 µM Latrunculin B (BIOMOL International L.P, Plymouth Meeting, PA); 0.1 and 10 µM Nocodazole (BIOMOL International L.P.); 0.5 and 1.0 µM Jasplakinolide (Invitrogen, Carlsbad, CA). The GFP-actin construct was provided by Bernhard Wehrle-Haller (Geneva, Switzerland) [59].

### Fixation and Permeabilization of NKL Cells

Perforin staining in NK cells was described [11]. Briefly, 1×10^6^ NKL cells were fixed with 4% freshly prepared paraformaldehyde for 15–30 minutes at room temperature (RT), washed with PBS for 3 times. Cells were permeabilized in 0.5% Triton-X100 and 10% normal donkey serum (NDS) in PBS for 30 minutes at RT. Cells were stained with anti perforin monoclonal antibody mouse IgG2b (Clone: δG9, Pierce Chemical Co. Rockford, IL, USA) for 60 minutes at RT. The primary antibody was diluted with 0.05% Triton-X100 and 3% NDS in PBS (1∶333 dilution). After three washes in PBS, cells were incubated for 1 hour at RT with appropriate secondary antibodies in 0.05% Triton-X100 and 3% NDS in PBS. Secondary antibody used was Alexa Fluor 647 conjugated goat anti-mouse IgG2b (1∶1000 dilution) (Molecular Probes, Eugene, OR, USA).

### Mouse NK Cells

Wild-type C3H/HeSnJ (C3H) mice were purchased from Taconic (Rockville, MD). *Ashen* mice were a gift of John A. Hammer (National Heart, Lung, and Blood Institute, National Institutes of Health, Bethesda, MD). Mice were maintained in the Comparative Medicine Branch of the NIAID, NIH (Twinbrook II Facility, Rockville, MD, USA). All animal protocols were approved by the NIAID Animal Care and Use Committee. The animal protocols number for this study is LIG-32. Unless otherwise specified, anti-mouse surface molecule antibodies were purchased from BD PharMingen. The antibodies were as follows: CD3 (clone, 145-2C11, Cat. No. 553062); CD49b (clone, KMC8, Cat. No. 553758); CD16 (clone, 2.4G2, Cat. No. 553144); NK1.1 (clone, PK136, Cat. No. 557391). NK cells were isolated from mouse spleen by negative selection with an NK isolation kit (Miltenyi Biotec, Auburn, CA), and were >90% CD3^−^, NK1.1^+^, CD49b^+^ (DX5^+^), and CD16^+^. Briefly, spleen cells were treated in ACK lysing buffer (Cat No. 304–000, Biofluids) to lyse erythrocytes for 5 minutes at room temperature. Cells were washed three times in RPMI 1640 (Gibco, Grand Island, NY, USA), and resuspended in RPMI 1640. Cells were negatively selected by passing through a MACS column. Freshly isolated mouse NK cells were resuspended in RPMI 1640 containing 10% fetal calf serum, 1% L-glutamine, 1% sodium pyruvate, 50 µM 2-mercaptoethanol (Sigma-Aldrich) and 800 U/ml of recombinant IL-2 (Hoffmann-La Roche).

### Rab27a shRNA

A plasmid containing Rab27a shRNA (Mission shRNA plasmid DNA, pLKO.1-Puro lentiviral vector) was obtained from Sigma (St. Louis, MO). The Rab27a shRNA targeting the sequence 5′-GGCAGGAGAGGTTTCGTAGCTTA- 3′ in the 5′ CDS of Rab27a gene was inserted into the pLKO.1-Puro lentiviral vector. A stable Rab27a shRNA clone was obtained by transfecting NKL-GFP-FasL cells with the Rab27a shRNA plasmid DNA. 72 hours after transfection, 0.5 µg/ml puromycin was added, and increased to 1.0 µg/ml after another 48 hours. Transfected cells were subcloned to obtain a stable NKL-GFP-FasL-Rab27a-shRNA cell line. Rab27a expression was assessed by Western blot and quantified with the Odyssey Infrared imaging system. The anti-human Rab27a antibody (Cat. No. ab55667) was purchased from Abcam (Cambridge, MA).

### Image acquisition

Spinning disc confocal and TIRF imaging were performed on an Olympus inverted IX-81 microscope, ASI MS-2000 controller (Applied Scientific Instrumentation, Inc., Eugene, OR, USA) motorized stage for xyz movements, electron-multiplier charge-coupled devices (Photometrics QuantEM, Roper Scientific), Olympus TIRF module, and lasers launched in a single mode fiber via an AOTF (NEOS Technologies, Melbourne, Florida, USA). The 100×1.45 N.A. objective from Zeiss (Zeiss, ∂ plan-Fluar 100x/1.45 oil) was used for all experiments. Illumination was provided by the 488 nm line of an Argon laser (150 W, Laser Physics, Salt Lake City, UT, USA). The hardware on the microscope was controlled by Metamorph software (Downingtown, PA). TIRF images were recorded at 100 ms/frame. 3D image stacks with 40 single frames (25 ms/frame) and a total axial dimension of 8 µm were recorded at 1 sec/stack.

### Three-dimensional tracking of LG

For three-dimensional particle tracking, Imaris 6.1 software (Bitplane AG, Zurich, Switzerland) was used. The estimated diameter for structures to be tracked was set to 0.3 µm, in accordance with the lateral optical resolution of our setup. Autoregressive motion was selected as the tracking algorithm. The parameters “MaxDistance” and “MaxGapSize” of the software were set to 0.5 and 2 µm, respectively. LG within a distance of 0.2 µm to the PM have been removed by selecting a 3D region of interest using Imaris 6.1 software. First, the PM is identified by superimposing maximum intensity projections of image stacks to outline the cell surface. Second, the region of interest, i.e. the cell interior, is selected in all x, y, and z directions. Tracks with a least 6 recorded steps were kept for further analysis.

### Three dimensional track analysis

To quantify the mobility of single LG, we calculated diffusion coefficients (D) based on the least-square fits of the functions described below to the mean square displacement (MSD) as a function of time (MSD(Δt)) [26], [60]. We defined five classes of motion, to characterize the mobility of each LG: *random movement* (MSD(Δt) is a straight line), *directed motion* superimposed on diffusion (MSD(Δt) deviates from a straight line and shows an upward curvature), and *restricted movement in a ‘cage’* (MSD(Δt) shows a downward curvature). In addition, we defined conditions for which a LG was assigned to be *immobile*. Finally, if a track did not meet any of the above conditions within a certain error, its movement was considered “complex”. To quantify the motion of the LG, we used the following equations to fit the MSD data: random diffusion with a diffusion coefficient D was fitted with
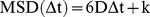
(1)directed diffusion at velocity v and with a diffusion coefficient D was fitted with:
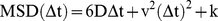
(2)and restricted diffusion was fitted with
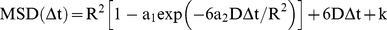
(3)where R is the radius of the cage, D is the diffusion coefficient of the cage, and a_1_≈0.99, a_2_≈0.85 are constants [26], [36]. In all equations, k is the constant offset of the MSD at all time steps [61], which is caused by the limited positional accuracy with which the center of LG has been determined by means of calculating the center of mass in 3D. The positional accuracy is mainly caused by the noise found in the recorded images.

### Sorting different types of movement

To sort trajectories into the five categories of movement types (i.e. immobile, random, directed, caged, and complex), we first defined the maximum MSD value of a track for which we still considered a vesicle to be immobile. This value was based on the tracking of fixed LG: 95% of all LG recorded in the NKL-GFP-FasL fixed by 4% freshly prepared paraformaldehyde for 15–30 minutes at room temperature, were found to have a maximum MSD value of 0.2 µm^2^. Hence, all tracked LG, which never exceed this MSD value within the total observation time of 50 s, were defined as “immobile”.

To assign the remaining types of movements to the trajectories we fitted equations 1–3 to the MSDs and calculated the respective values of R^2^ for each fit (R^2^, correlation coefficient, indicating the strength and direction of a linear relationship between two random variables). In general, a track was assigned to the type of movement, which achieved the highest R^2^, i.e. the best fit. If R^2^ was below 0.33 for all fits, we defined the movement as “complex”, to indicate, that it was not possible to mathematically describe it with one of the three basic types of diffusion. This was, however, never the case for more than 5% of analyzed trajectories.

If R^2^ of the MSD fit to equation 2 was larger than fits to equation 1 or 3, the LG movement type “directed” was assigned. If the fit of the MSD to equation 3 achieved the highest R^2^, the LG was classified as showing “caged” movement. If equation 1 resulted in the highest value for R^2^, LG were considered to have “random” movement. In addition, if the standard deviation of all three R^2^ values for the fits to the equations for random, directed, and caged movement was below 0.015, movement was also considered as “random”. This approach was necessary to ensure that trajectories with a nearly linear MSD were not assigned to either directed or caged movements, because these equations have more parameters than the equation for random movement. More parameters generally result in a lower value for R^2^.

### Two - dimensional tracking analysis

Essentially, the same approach was used to analyze 2D trajectories obtained from TIRF microscopy measurements. For tracking vesicles in 2D, we used MatLab algorithms adapted by Daniel Blair and Eric Dufresne (http://physics.georgetown.edu/matlab/) from particle tracking procedures originally written for IDL by David Grier, John Crocker, and Eric Weeks. In this case, however, random diffusion with a diffusion coefficient D is described by the equation
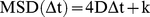
(4)directed diffusion at velocity v with a diffusion coefficient D is described by the equation
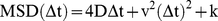
(5)and restricted diffusion within a cage of radius R and a diffusion coefficient of D_0_ is described by equation [62]

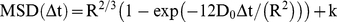
(6)


In all equations, k is the constant offset of the MSD at all time steps [61]. More than 95% of the LG recorded in the NKL-GFP-FasL fixed by 4% freshly-prepared paraformaldehyde for 15–30 minutes at room temperature, were found to have a maximum MSD value of 0.2 µm^2^. Hence, all tracked LG, which never exceed this MSD value within the total observation time of 50s, were defined as “immobile”.

### Track properties

To further quantify the movement of LG, the length, straightness, and displacement were calculated for each track. For a multi-step trajectory its length is defined as the sum of all step-displacements, its total displacement is the displacement between the start and end of the track and its straightness is defined as the ratio between its total displacement and length. Lower straightness values correspond to constrained trajectories. Key parameters from the automated tracking analysis (i.e. length, displacement, straightness, and velocity) are presented as both histograms and cumulative probability distributions.

The cumulative probability distribution and histograms plot the same information for a given data set; however, the histogram plots the frequency distribution (y-axis) as a function of the binned data set (x-axis). The cumulative probability distribution displays the distribution of the data set from the smallest (from the left on the x-axis) to the greatest value (at the right of the x-axis) and provides the probability (y-axis) of whether a particular value will occur at or less than a specified point on the x-axis. Moreover, the cumulative probability distributions are less sensitive to noise and they are continuous, i.e. bin-sizes do not affect their appearance as they do in histogram plots. Since most readers are familiar with standard histogram plots, we present histogram plots (an overview of the distribution) and cumulative probability distributions (rapid identification of the median value of the data set by interpolation at 50% on the y-axis).

### Statistical analysis

For normally distributed data, averages were given as mean ± SEM (standard error of the mean) and statistical significance was tested using the Student's t-test. For exponential distribution data, averages were given as median ± SEM and statistical significance was tested using the Kolmogorov-Smirnov test.

## Supporting Information

Figure S1 Disruption of the cytoskeleton decreased the mobility of LG at the PM. 2D track length, displacement, and straightness in NKL cells either untreated (Control) or treated with 10 µM Nocod (A), 10 µM LatB (B), or both (C).
(0.71 MB TIF)

Figure S2 Disruption of the cytoskeleton decreased the fraction of LG with directed movement and increased the fraction of immobile LG. The relative occurrence of each type of movement for each indicated condition is summarized. The numbers of tracking events (n) are listed on the right.
(0.25 MB TIF)

Figure S3 Long-range directed movement of single LG depended on microtubule, but not F-actin and Rab27a. Representative single LG tracked among different conditions, as indicated. An accumulated view of trajectories recorded during ∼50 seconds is shown. All trajectories are centered with their initial time point in the middle of each figure. Note the extension of the cloud of tracks obtained from measurements of fixed granules provides a conservative estimate for the spatial resolution achieved by the tracking method in all three dimensions.
(0.96 MB TIF)

Figure S4 Stabilization of F-actin and disruption of microtubules impaired cytosolic LG movement. Straightness of trajectories is shown as histograms and cumulative probability plots, as observed after treatment with 10 µM LatB (A), 1.0 µM Jasp (B), and 10 µM Nocod (C). Images were acquired by 3-D spinning disc confocal microscopy.
(0.76 MB TIF)

Figure S5 High concentration of LatB slightly increased the 3D tracks length of LG in the cytosol. 3D length of tracking is shown as histograms (A) and cumulative probability plots (B), as observed after treatment with DMSO vehicle control (black line), 1.0 µM LatB (blue line), and 10 µM LatB (red line). Images were acquired by 3-D spinning disc confocal microscopy.
(0.29 MB TIF)

Figure S6 Disruption of microtubules decreased the mobility of cytosolic LG. The length of trajectories of vesicles observed in NKL cells pretreated with 10 µM Nocod are shown as histograms (A) and cumulative probability plots (B). For the same trajectories, displacement (C and D) and velocity (E and F) are shown as histograms and cumulative probability plots, respectively.
(0.66 MB TIF)

Figure S7 Data from additional two independent Rab27a knockdown NKL clones are comparable. The tracks length of single vesicle trajectories observed in Rab27a knockdown (Rab27a-KD-A1) NKL cells (red), NKL cells expressing control shRNA (control, black), as well as fixed cells (gray) are shown as histograms (A) and cumulative probability plots (B). The straightness of single trajectories during 10 seconds in NK cells from Rab27a-KD-A1 (red) and control shRNA (black) were compared using histograms (C) and cumulative probability plots (D). The relative occurrence of each type of movement for each indicated condition is summarized in a bar plot (E). The numbers of tracking events (n) are listed on the right. The data are representative of at least two independent experiments.
(0.82 MB TIF)

Table S1 Rab27a decreases the velocity of the directed movement of LG. aVelocity in µm/s. bNKL cells expressing GFP-FasL. cMouse NK cells labeled with LysoTracker Red DND-99.
(0.04 MB DOC)

Video S1 Rab27a Defect Reduces the Number of LG at the Plasma Membrane. Time-lapsed TIRF imaging of LG in NK cells. Left panel: representative TIRF image of paraformaldehyde-fixed NKL-GFP-FasL cells. Middle panel: representative TIRF image of untreated NKL-GFP-FasL cells. Right panel: representative TIRF image of Rab27a-KD in NKL-GFP-FasL cells. Images were acquired at 100 ms/frame. Scale bar represents 3.0 µm.
(8.23 MB MOV)

Video S2 3-D Tracking of LG in a Live Untreated NK Cell by High-speed Spinning Disc Confocal Microscopy. LG in an untreated NKL-GFP-FasL cell are shown in Green. Images were acquired for 20 time points of about 1 second each. Each time point contains 40 axial steps of 0.2 micron in the z - direction. Each grid represents 1.0 µm.
(0.79 MB MOV)

Video S3 3-D Tracking of LG in Live NK Cell Treated with LatB by High-speed Spinning Disc Confocal Microscopy. Time-lapsed 3D spinning disc confocal microscopy of live NKL-GFP-FasL cells. LG in NKL-GFP-FasL cells treated with 10 µM LatB are shown in Green. Images were acquired for 20 time points of about 1 second each. Each time point contains 40 axial steps of 0.2 micron in the z - direction. Each grid represents 1.0 µm.
(1.63 MB MOV)

Video S4 3-D Tracking of LG in a Live NK Cell Treated with Nocod by High-speed Spinning Disc Confocal Microscopy. Time-lapsed 3D spinning disc confocal microscopy of live NKL-GFP-FasL cells. LG in NKL-GFP-FasL cells treated with 10 µM Nocod are shown in Green. Images were acquired for 20 time points of about 1 second each. Each time point contains 40 axial steps of 0.2 micron in the z - direction. Each grid represents 1.0 µm.
(0.92 MB MOV)

Video S5 The Distribution of Actin and Perforin Containing LG by 3D Confocal Microscopy. Serial confocal images taken through an NKL cell bound to a poly-L-lysine coated coverslip with DMSO vehicle control. Actin and perforin are shown in Green and Red, respectively. Perforin-containing lytic granules are not directly surrounded by accumulated F-actin. The signal from F-actin is weak.
(8.24 MB MOV)

Video S6 A Dense Actin Meshwork Restricts LG in NKL cells with Jasp treatment. Serial confocal images taken through one NKL cell bound to a poly-L-lysine coated coverslip with 1.0 µM Jasp treatment. Actin and perforin are shown in Green and Red, respectively. Perforin-containing granules are directly caged by accumulated F-actin. The signal from F-actin is strong.
(4.28 MB MOV)

Video S7 3-D Tracking of LG in Live Rab27a-KD NKL-GFP-FasL Cell by High-speed Spinning Disc Confocal Microscopy. Time-lapsed 3D spinning disc confocal microscopy of live NKL-GFP-FasL cells. LG in Rab27a-KD in NKL-GFP-FasL cells are shown in Green. Images were acquired for 20 time points of about 1 second each. Each time point contains 40 axial steps of 0.2 micron in the z - direction. Each grid represents 1.0 µm.
(1.28 MB MOV)
