# Supplementary figures and images for: Distinct Role of Rab27a in Granule Movement at the Plasma Membrane and in the Cytosol of NK Cells
Source: PLoS One. 2010 Sep 21;5(9):e12870. doi: 10.1371/journal.pone.0012870 (PMC2943471; doi:10.1371/journal.pone.0012870)

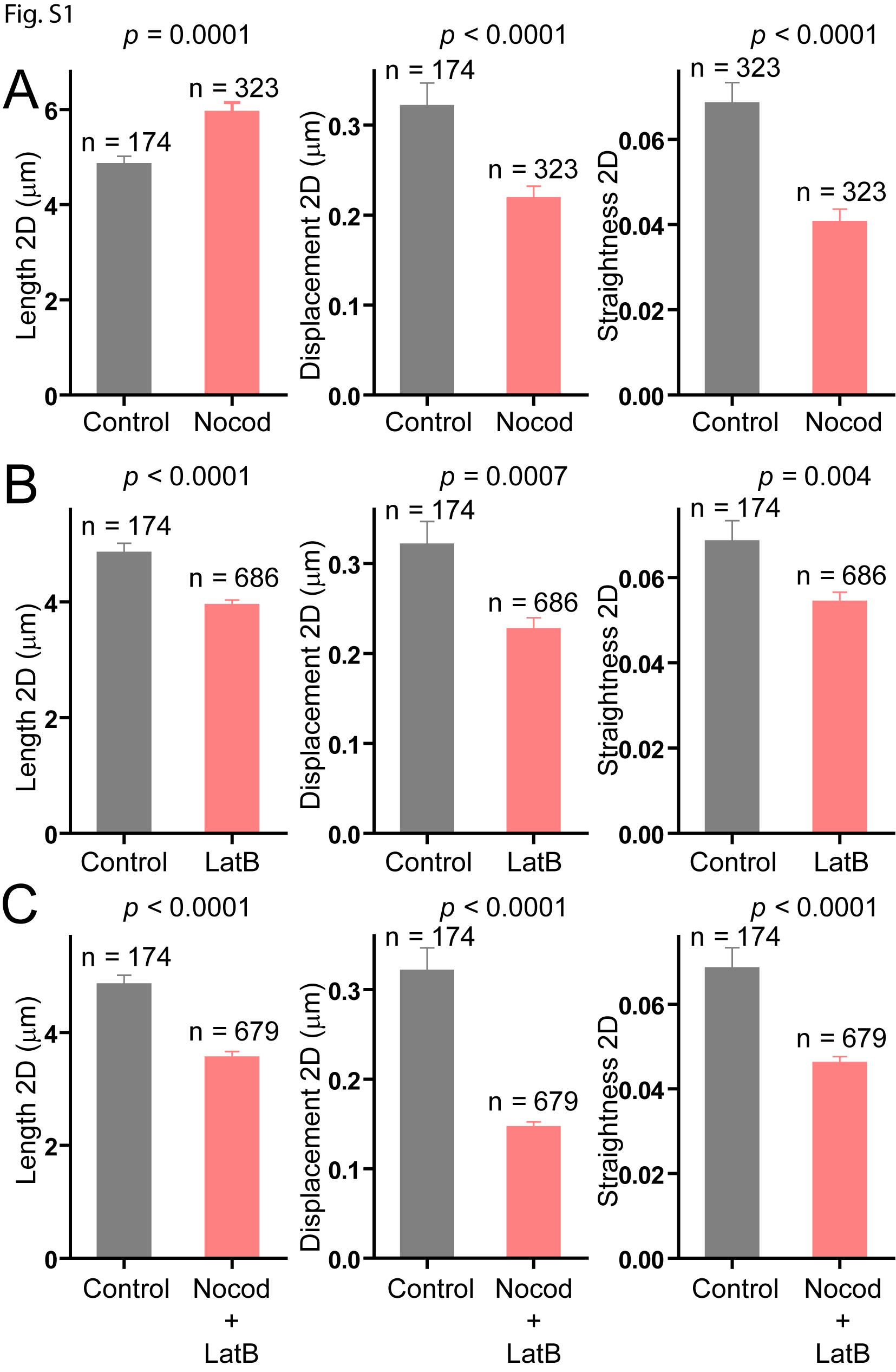

Supplement: Figure S1 — Disruption of the cytoskeleton decreased the mobility of LG at the PM. 2D track length, displacement, and straightness in NKL cells either untreated (Control) or treated with 10 µM Nocod (A), 10 µM LatB (B), or both (C). (0.71 MB TIF) [file pone.0012870.s001.tif]

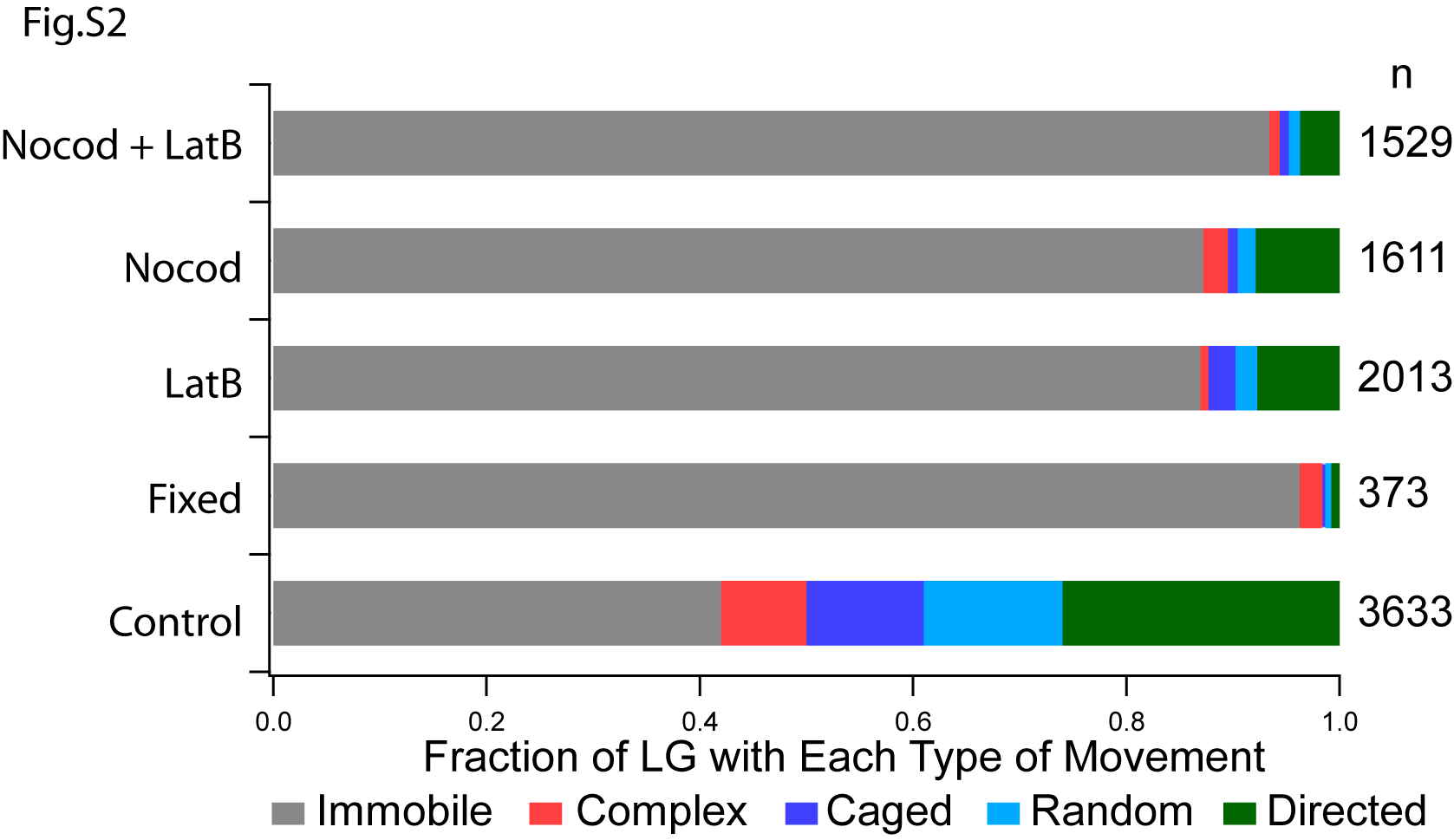

Supplement: Figure S2 — Disruption of the cytoskeleton decreased the fraction of LG with directed movement and increased the fraction of immobile LG. The relative occurrence of each type of movement for each indicated condition is summarized. The numbers of tracking events (n) are listed on the right. (0.25 MB TIF) [file pone.0012870.s002.tif]

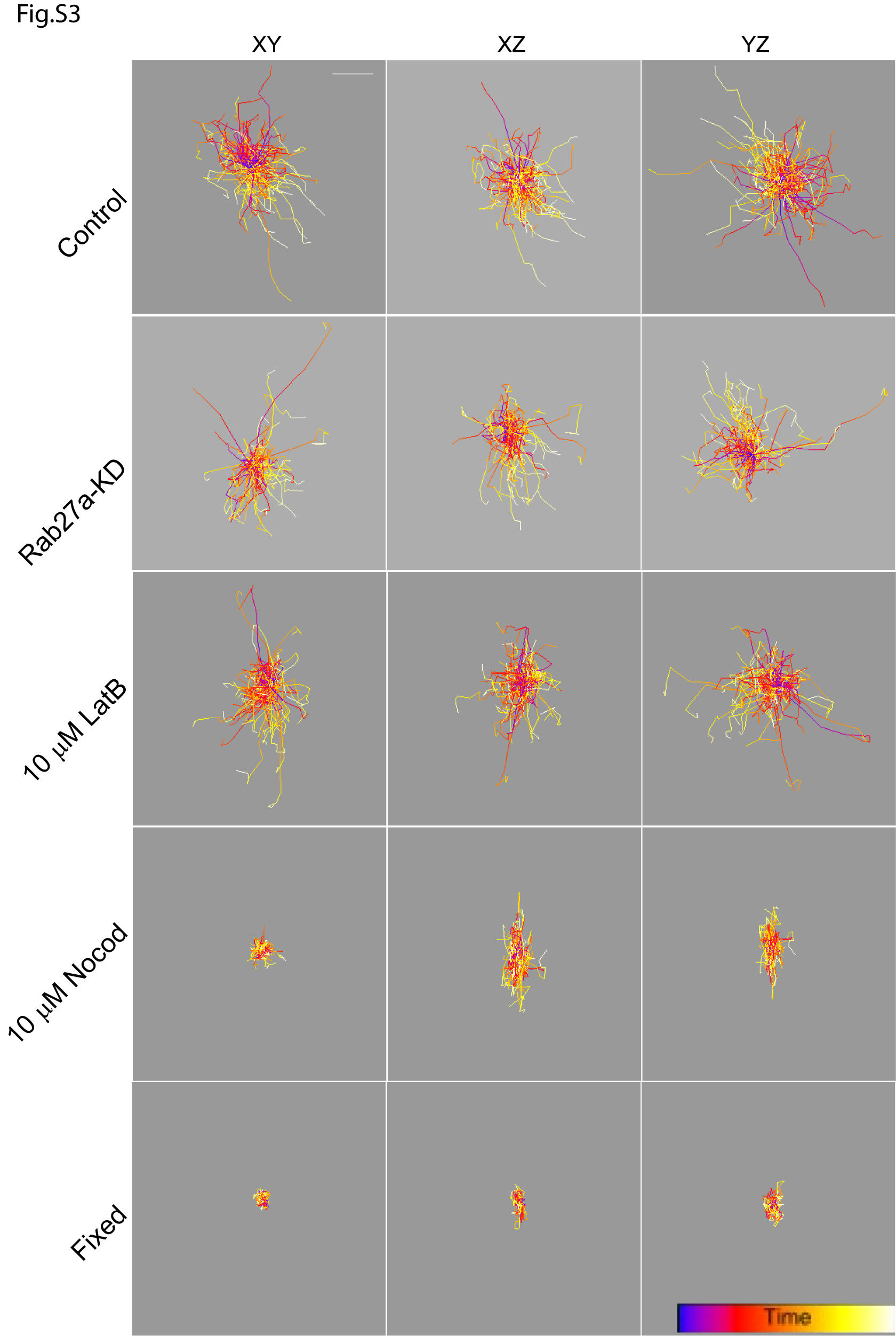

Supplement: Figure S3 — Long-range directed movement of single LG depended on microtubule, but not F-actin and Rab27a. Representative single LG tracked among different conditions, as indicated. An accumulated view of trajectories recorded during ∼50 seconds is shown. All trajectories are centered with their initial time point in the middle of each figure. Note the extension of the cloud of tracks obtained from measurements of fixed granules provides a conservative estimate for the spatial resolution achieved by the tracking method in all three dimensions. (0.96 MB TIF) [file pone.0012870.s003.tif]

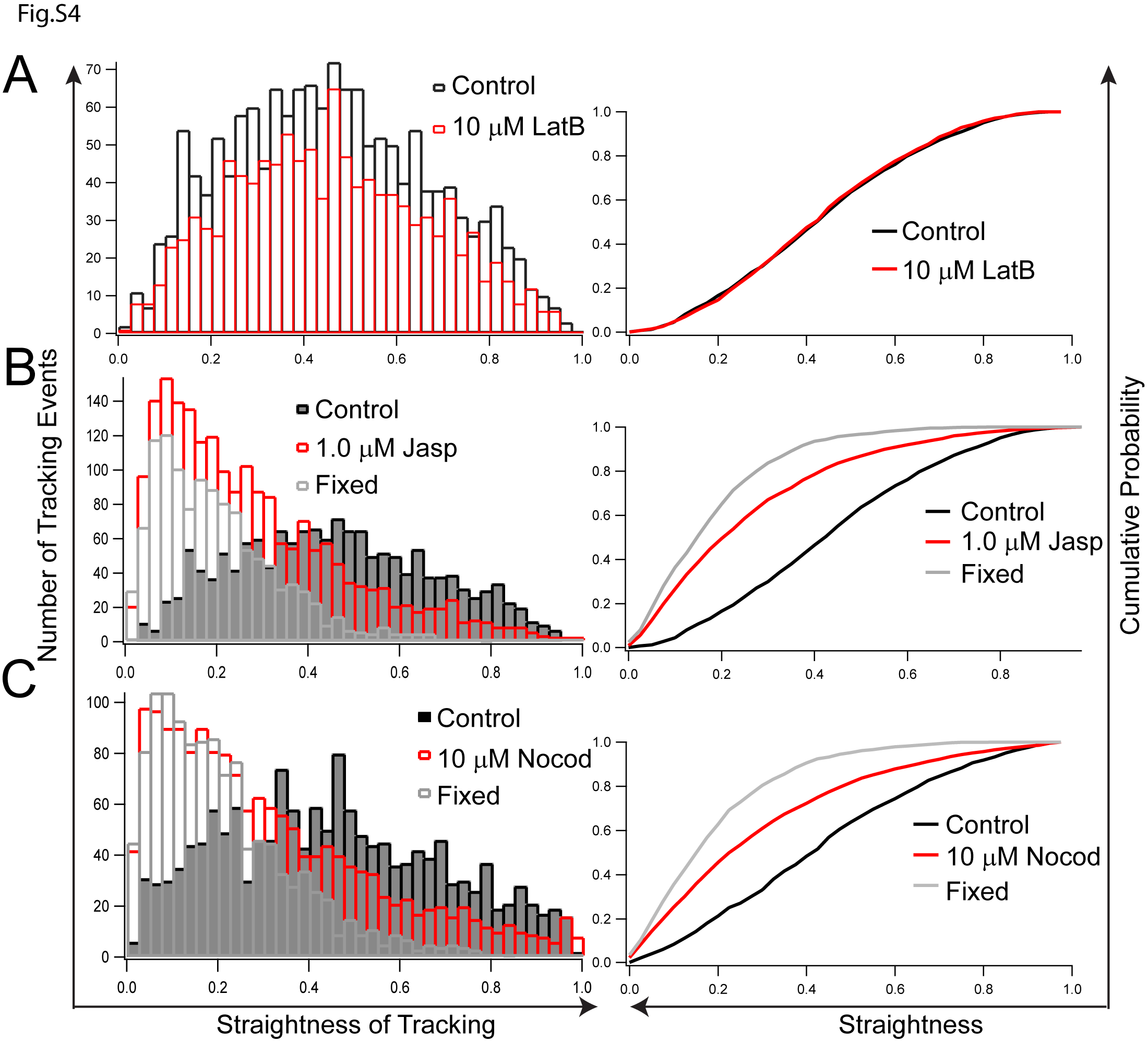

Supplement: Figure S4 — Stabilization of F-actin and disruption of microtubules impaired cytosolic LG movement. Straightness of trajectories is shown as histograms and cumulative probability plots, as observed after treatment with 10 µM LatB (A), 1.0 µM Jasp (B), and 10 µM Nocod (C). Images were acquired by 3-D spinning disc confocal microscopy. (0.76 MB TIF) [file pone.0012870.s004.tif]

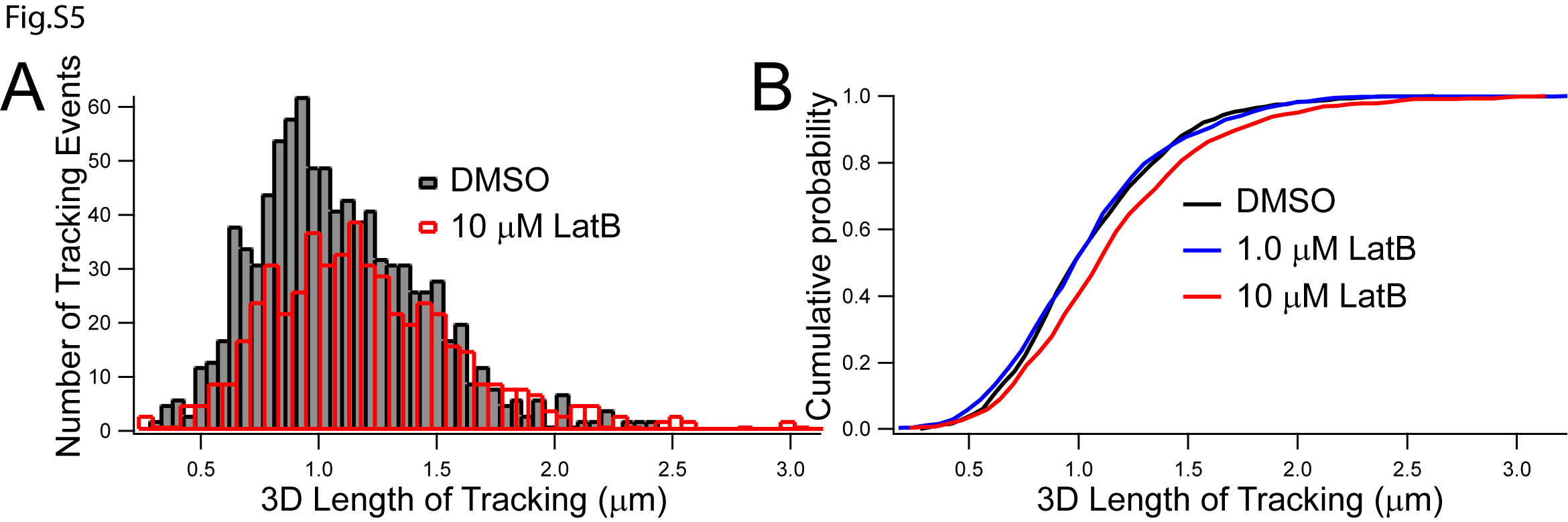

Supplement: Figure S5 — High concentration of LatB slightly increased the 3D tracks length of LG in the cytosol. 3D length of tracking is shown as histograms (A) and cumulative probability plots (B), as observed after treatment with DMSO vehicle control (black line), 1.0 µM LatB (blue line), and 10 µM LatB (red line). Images were acquired by 3-D spinning disc confocal microscopy. (0.29 MB TIF) [file pone.0012870.s005.tif]

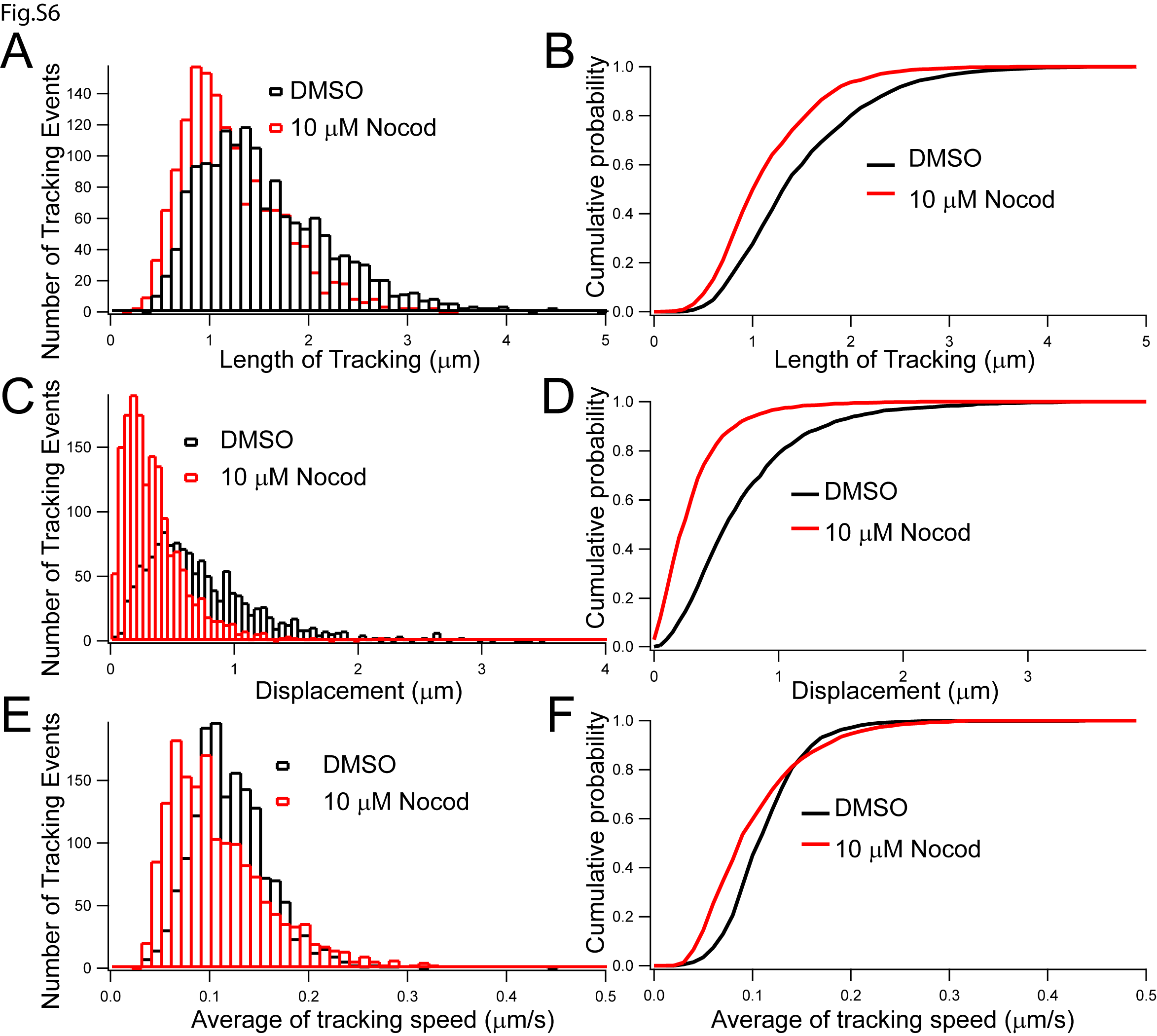

Supplement: Figure S6 — Disruption of microtubules decreased the mobility of cytosolic LG. The length of trajectories of vesicles observed in NKL cells pretreated with 10 µM Nocod are shown as histograms (A) and cumulative probability plots (B). For the same trajectories, displacement (C and D) and velocity (E and F) are shown as histograms and cumulative probability plots, respectively. (0.66 MB TIF) [file pone.0012870.s006.tif]

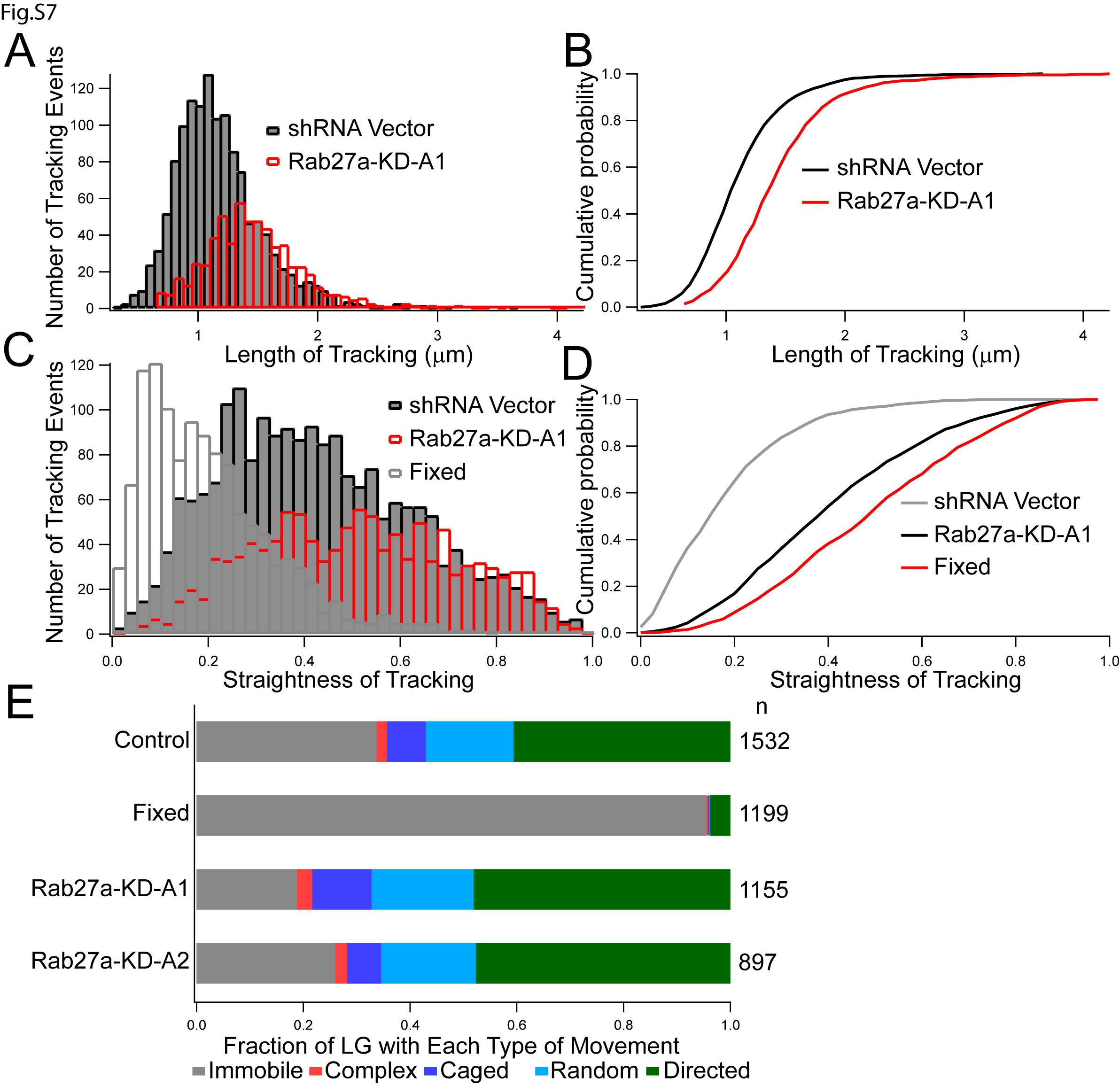

Supplement: Figure S7 — Data from additional two independent Rab27a knockdown NKL clones are comparable. The tracks length of single vesicle trajectories observed in Rab27a knockdown (Rab27a-KD-A1) NKL cells (red), NKL cells expressing control shRNA (control, black), as well as fixed cells (gray) are shown as histograms (A) and cumulative probability plots (B). The straightness of single trajectories during 10 seconds in NK cells from Rab27a-KD-A1 (red) and control shRNA (black) were compared using histograms (C) and cumulative probability plots (D). The relative occurrence of each type of movement for each indicated condition is summarized in a bar plot (E). The numbers of tracking events (n) are listed on the right. The data are representative of at least two independent experiments. (0.82 MB TIF) [file pone.0012870.s007.tif]
