# Supplementary material for: Distinct Role of Rab27a in Granule Movement at the Plasma Membrane and in the Cytosol of NK Cells
Source: PLoS One. 2010 Sep 21;5(9):e12870. doi: 10.1371/journal.pone.0012870 (PMC2943471; doi:10.1371/journal.pone.0012870)
Supplement: Table S1 — Rab27a decreases the velocity of the directed movement of LG. aVelocity in µm/s. bNKL cells expressing GFP-FasL. cMouse NK cells labeled with LysoTracker Red DND-99. (0.04 MB DOC) [file pone.0012870.s008.doc]

**Supplementary Table S1**

**Table S1.** Rab27a decreases the velocity of the directed movement of LG.

| **Microscopy** | **Cell** | **Phenotype** | **Mean^a^ ± SEM (n)** | ***p* value** |
| --- | --- | --- | --- | --- |
| 2D | NKL^b^ | shRNA control | 0.30 ± 0.01 (602) |  |
|  |  | Rab27-KD | 0.38 ± 0.02 (328) | 0.015 |
|  | Mouse NK^c^ | C3H | 0.15 ± 0.01 (250) |  |
|  |  | *Ashen* | 0.24 ± 0.02 (164) | 0.001 |
| 3D | NKL | shRNA control | 0.07 ± 0.001 (647) |  |
|  |  | Rab27-KD | 0.11 ± 0.001 (940) | 0.0001 |
|  | Mouse NK | C3H | 0.08 ± 0.004 (97) |  |
|  |  | *Ashen* | 0.11 ± 0.005 (128) | 0.0001 |

^a^Velocity in µm/s.

^b^NKL cells expressing GFP-FasL.

^c^Mouse NK cells labeled with LysoTracker Red DND-99.
